# Arrest defective 1 regulates the oxidative stress response in human cells and mice by acetylating methionine sulfoxide reductase A

**DOI:** 10.1038/cddis.2014.456

**Published:** 2014-10-23

**Authors:** S-H Shin, H Yoon, Y-S Chun, H-W Shin, M-N Lee, G T Oh, J-W Park

**Affiliations:** 1 Department of Biomedical Science, Seoul National University College of Medicine, Seoul, Korea; 2 Department of Pharmacology, Seoul National University College of Medicine, Seoul, Korea; 3 Ischemic/Hypoxic disease Institute, Seoul National University College of Medicine, Seoul, Korea; 4 Department of Life Science, Ewha Womans University, Seoul, Korea

## Abstract

Methionine sulfoxide reductase A (MSRA) protects proteins from oxidation, and also helps remove reactive oxygen species (ROS) by recovering antioxidant enzymes inactivated by oxidation. Although its functions have been investigated extensively, little is known about the mechanism by which MSRA is regulated. Arrest defective 1 (ARD1) is an enzyme that catalyzes not only N-terminal acetylation as a cotranslational modification but also lysine acetylation as a posttranslational modification. ARD1, which is expressed in most cell types, is believed to participate in diverse biological processes, but its roles are poorly understood. Given that MSRA was hunted in a yeast two-hybrid screen with ARD1 as the bait, we here investigated whether ARD1 is a novel regulator of MSRA. ARD1 was shown to interact with and acetylate MSRA in both cells and test tubes. It specifically acetylated the K49 residue of MSRA, and by doing so repressed the enzymatic function of MSRA. ARD1 increased cellular levels of ROS, carbonylated proteins and DNA breaks under oxidative stress. Moreover, it promoted cell death induced by pro-oxidants, which was attenuated in MSRA-deficient cells. When mice were exposed to hyperoxic conditions for 2 days, their livers and kidneys were injured and protein carbonylation was increased. The oxidative tissue injury was more severe in ARD1 transgenic mice than in their wild-type littermates. In conclusion, ARD1 has a crucial role in the cellular response to oxidative stress as a bona fide regulator of MSRA. ARD1 is a potential target for ameliorating oxidative injury or for potentiating ROS-producing anticancer agents.

Aerobic respiration is essential for eukaryotic life because molecular oxygen participates in ATP production and various oxidative metabolic reactions.^1^ When oxygen is used, reactive oxygen species (ROS) are inevitably generated and threaten life as harmful metabolites that damage macromolecules such as nucleic acids, lipids and proteins.^2,3^ ROS also act as second messengers that promote cell proliferation or differentiation.^4, 5, 6, 7^ From a functional perspective, ROS act as a double-edged sword in determining cell fate, and the roles of ROS depend on cell contexts.^8^ A variety of cell metabolic reactions are regulated depending on the intracellular redox state, which reflects the balance between ROS-generating oxidases and ROS-scavenging antioxidants.^9^ Accordingly, knowledge about the redox-balancing mechanism will help us to better understand normal physiology and pathology.

The sulfur atom of methionine is easily oxidized by ROS, with methionine being modified to methionine sulfoxide (MetO), which forms two enantiomers (*S*-sulfoxide and *R*-sulfoxide).^10^ When proteins are sulfoxidized at methionine residues, their functions become impaired or altered.^11^ Therefore, MetO is not only a convincing biomarker for reflecting the extent of oxidative stress but also a pathogenic factor that contributes to oxidative stress-related diseases.^12^ As MetO causes serious problems in life, the defense systems against MetO have been evolutionally conserved in prokaryotic and eukaryotic cells.^13^ One such system, methionine sulfoxide reductase (MSR), has a crucial role in preventing the accumulation of MetO, and includes two enzymes, methionine sulfoxide reductase A (MSRA) and MSRB, which reduce *S*-sulfoxide and *R*-sulfoxide, respectively.^14^

Arrest defective 1 (ARD1) is an enzyme that catalyzes N-terminal acetylation of nascent peptides as a cotranslational modification and lysine acetylation as a posttranslational modification.^15^ In yeast and mammalian cells, ARD1 is known to have essential roles in cell growth and differentiation.^16,17^ ARD1 has also been reported to control cell migration by acetylating myosin light chain kinase^18^ and to promote cancer growth by acetylating *β*-catenin or the androgen receptor.^19^ Considering that ARD1 is widely expressed in most mammalian cells,^20^ it is expected that ARD1 has diverse functions beyond those mentioned above. To further understand the functions of ARD1, we sought novel targets of ARD1 using the yeast two-hybrid method and identified MSRA as an ARD1-interacting molecule. Furthermore, we tested the possibility that ARD1 determines cell fate under oxidative stress by regulating MSRA. This study may provide new insights into how MSRA is regulated and identifies ARD1 as a potential target for modulating the cellular response to oxidative stress.

## Results

### ARD1 associates with MSRA

**Figure 1:**
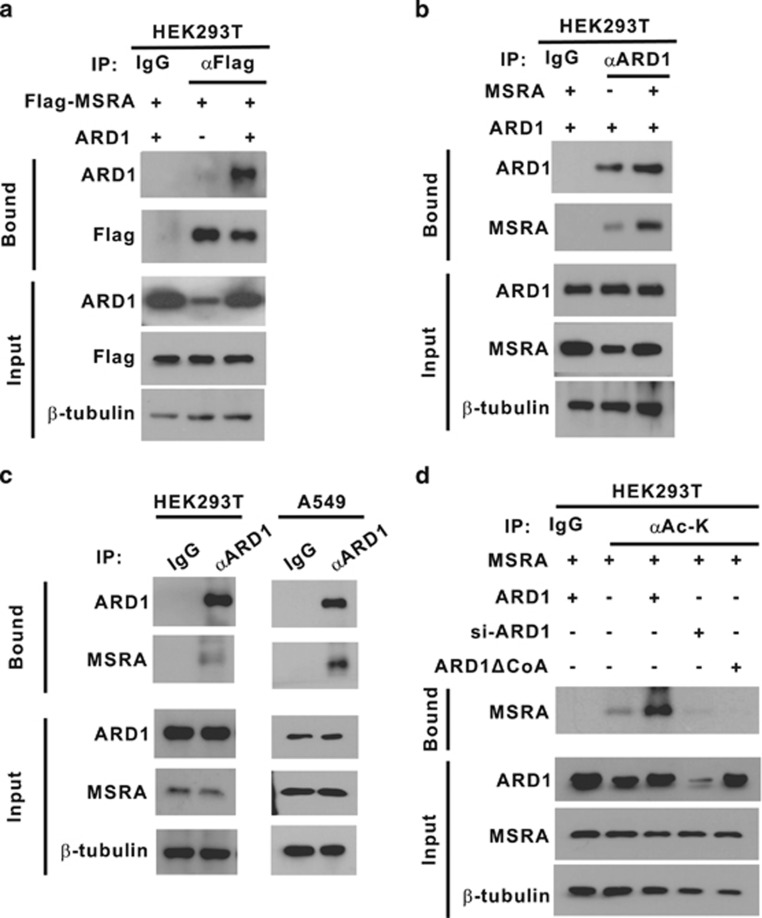
ARD1 binds and acetylates MSRA in cells. (**a**) HEK293T cells were cotransfected with pARD1 and pFlag-MSRA (1 *μ*g per 60-mm dish). Proteins in the cell lysates were immunoprecipitated with anti-Flag beads or with non-immunized rabbit serum (IgG), and ARD1 and Flag-MSRA in precipitates were immunoblotted with the indicated antibodies. (**b**) In HEK293T cells, which had been cotransfected with ARD1 and MSRA plasmids, the cell lysates were subjected to immunoprecipitation and immunoblotting using the indicated antibodies. (**c**) HEK293T or A549 cells were lysed and the proteins were immunoprecipitated with anti-ARD1 or non-immunized rat serum (IgG). Precipitated ARD1 and MSRA were immunoblotted. (**d**) HEK293T cells were cotransfected with pMSRA, pARD1, pARD1ΔCoA and/or ARD1-targeting siRNA (40 nM). Proteins in the cell lysates were immunoprecipitated with anti-Ac-K antibody or IgG, and precipitated MSRA was immunoblotted

We screened a human liver cDNA library with a bait of full-length human ARD1 in the yeast two-hybrid system, as previously described.^18^ Of 17 positive clones harboring in-frame cDNAs, 4 clones were shown to harbor cDNAs encoding amino acids 126–235 or 132–235 of MSRA (NM_012331). A yeast colony selected with three reporters is representatively shown in Supplementary Figure S1. To confirm the interaction between ARD1 and MSRA, we coexpressed ARD1 and Flag-MSRA in HEK293T cells and found that ARD1 and Flag-MSRA were co-precipitated by an anti-Flag antibody (Figure 1a). This interaction was cross-checked by changing the antibodies used for immunoprecipitation and immunoblotting (Figure 1b). The association between endogenous proteins was examined in the HEK293T and A549 cell lines, both of which were shown to express both ARD1 and MSRA in a preliminary study. Both endogenous proteins were co-precipitated in both cell lines (Figure 1c). To examine whether ARD1 acetylates MSRA, we expressed MSRA with ARD1, an ARD1ΔCoA mutant lacking the acetyl-CoA binding domain, or an ARD1-targeting siRNA. As shown in Figure 1d, the lysyl acetylation of MSRA was stimulated by ARD1 overexpression, but was inhibited by ARD1 knockdown or ARD1ΔCoA expression.

### ARD1 acetylates MSRA directly *in vitro*

**Figure 2:**
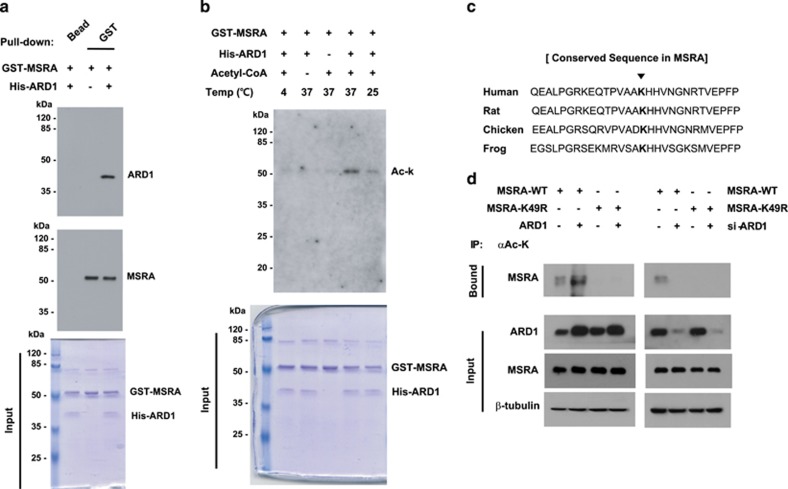
ARD1 binds and acetylates MSRA *in vitro*. (**a**) *In vitro* binding analysis. Recombinant His-ARD1 and GST-MSRA peptides, which had been isolated from *E*. *coli*, were put together in a test tube. His-ARD1 was pulled down using glutathione-affinity beads, and pulled-down proteins were immunoblotted. Input levels were verified by electrophoresis and Coomassie staining. (**b**) *In vitro* acetylation assay. Recombinant GST-MSRA was incubated with His-ARD1 and acetyl-CoA at 37, 25 or 4 °C. The lysine acetylation of GST-MSRA was identified using anti-Ac-K antibody, and input levels were verified by electrophoresis and Coomassie staining. (**c**) Conserved sequence of human, rat, chicken and flog MSRAs. (**d**) ARD1 acetylation of MSRA at K49. HEK293T cells were cotransfected with pMSRA or pMSRA-K49R and/or pARD1 or si-ARD1. Proteins in the cell lysates were immunoprecipitated with anti-Ac-K antibody and precipitated MSRA was immunoblotted

To test the possibility that ARD1 acetylates MSRA directly, we performed *in vitro* analyses using recombinant peptides. A GST pull-down assay revealed that His-ARD1 directly interacted with GST-MSRA (Figure 2a). Moreover, His-ARD1 lysyl acetylated GST-MSRA *in vitro* using acetyl-CoA, depending on the reaction temperature (Figure 2b). To determine which residue of MSRA is acetylated by ARD1, we separated digested peptides by liquid chromatography and analyzed the separated peptides by tandem mass spectrometry (LC-MS/MS). MSRA was shown to be acetylated at the K49 residue by ARD1, but not in the absence of ARD1 (Supplementary Figure S2). As shown in Figure 2c, the ARD1-acetylated motif is conserved among various species. To confirm the ARD1-catalyzed acetylation of K49, we constructed an MSRA-K49R mutant. As expected, wild-type MSRA in HEK293T cells was basally lysyl acetylated, and the acetylation was regulated ARD1-dependently. By contrast, MSRA-K49R was acetylated only weakly, even under conditions of ARD1 overexpression (Figure 2d). These findings indicate that ARD1 targets and acetylates MSRA at K49.

### ARD1 acts as a negative regulator of MSRA

**Figure 3:**
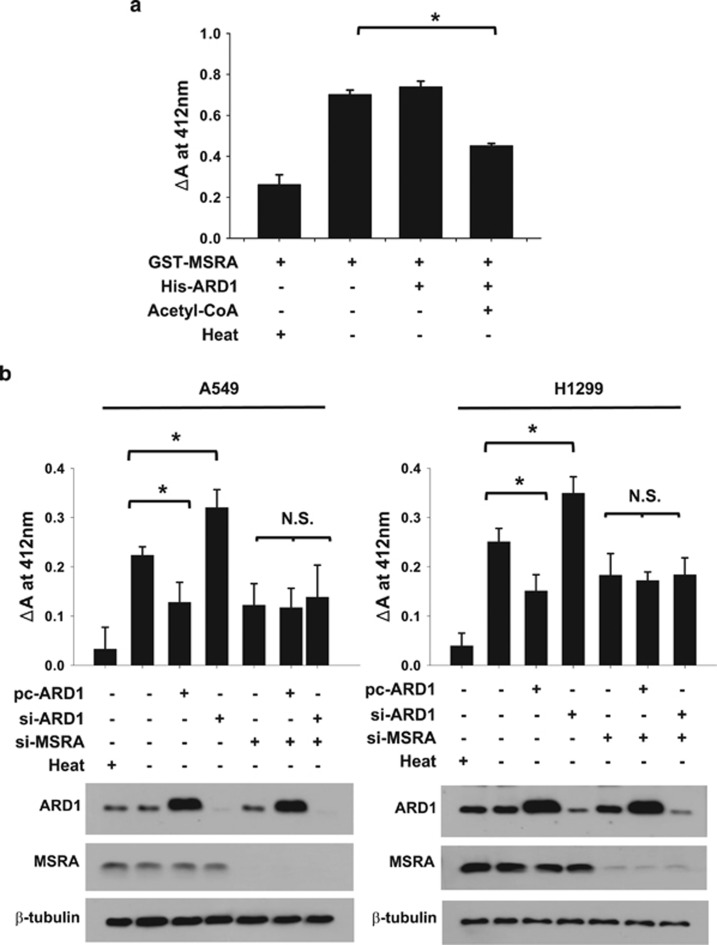
ARD1 inhibits MSRA activity. (**a**) *In vitro* assay for MSRA activity. MSRA activity was analyzed at 37 °C for 30 min in a reaction mixture containing 200 *μ*g of recombinant GST-MSRA, 100 *μ*g of recombinant His-ARD1 and/or 2 mM acetyl-CoA. The absorbance at 412 nm was changed along the enzymatic reaction by MSRA. Each bar represents the mean+S.D. from three independent experiments and *denotes *P*<0.01 between two groups. (**b**) The cell lysates from A549 or H1299 cells, which had been transfected with the indicated plasmid or siRNA, were at 37 °C for 30 min in the reaction buffer for MSRA. Each bar represents the mean+S.D. from three independent experiments. * and NS denote *P*<0.01 and *P*>0.05, respectively

To understand how ARD1 regulates MSRA, we analyzed the enzymatic activity of MSRA *in vitro* by measuring the absorbance change at 412 nm. Recombinant MSRA catalyzed the enzymatic reaction, which was abolished by brief heating. After MSRA was incubated with ARD1 and acetyl-CoA, its enzymatic activity decreased significantly (Figure 3a). We assessed MSRA activity in lysates from A549 and H1299 cells to further examine the inhibition of MSRA activity by ARD1. MSRA activity was found to be inhibited by ARD1 overexpression and increased by ARD1 knockdown in both cell lines (Figure 3b). When MSRA was knocked down, the absorbance change at 412 nm was reduced significantly, regardless of the ARD1 level, which confirms the specificity of this assay. On the basis of these results, we conclude that ARD1 is likely to inactivate MSRA through the acetylation of its K49 residue.

### ARD1 promotes cellular responses to oxidative stress

**Figure 4:**
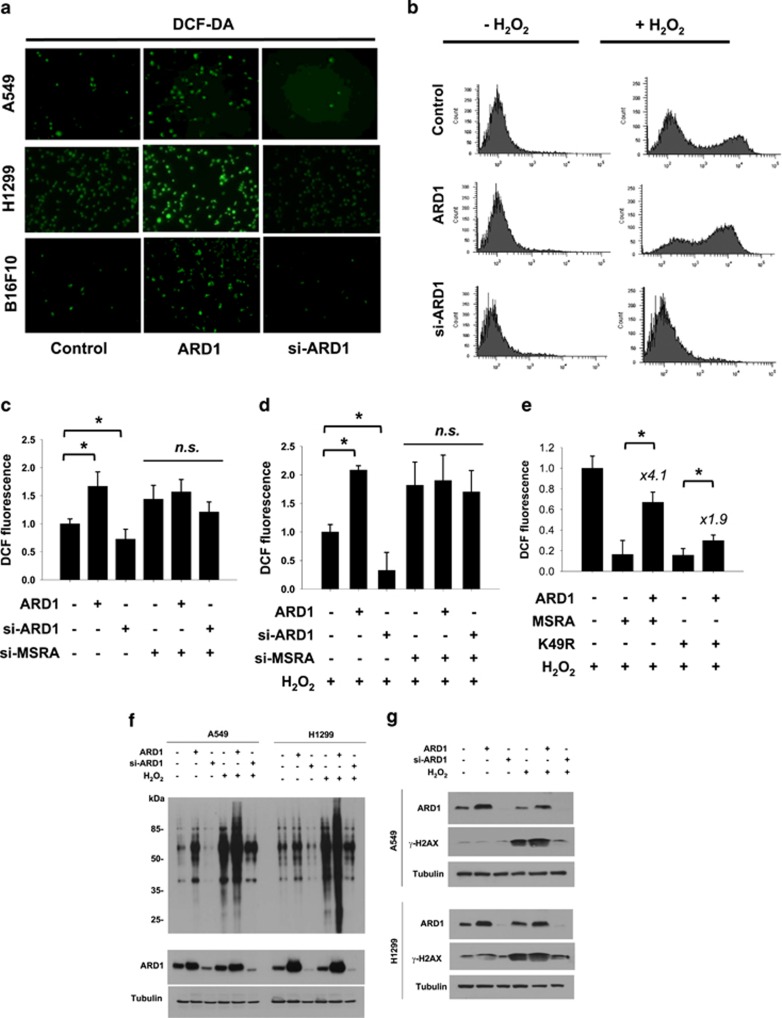
ARD1 increases oxidative stress in cells through its inhibition of MSRA. (**a**) Representative images of intracellular DCF fluorescence in A549, H1299 and B16F10 cells, which were transfected with ARD1 plasmid or siRNA. Cells were not subjected to additional oxidative stress. (**b**) A549 cells, which had been transfected with ARD1 plasmid or siRNA, were incubated with 20 *μ*M H_2_O_2_ or not for 30 min. Flow cytometric analyses were performed to detect cells emitting DCF fluorescence. (**c**–**e**) A549 cells, which had been transfected as indicated, were incubated with 20 *μ*M H_2_O_2_ or not for 30 min. On the basis of flow cytometric analyses, the total intensity of DCF fluorescence in each group was measured and presented as a relative value to the control. Results are shown as means+S.D.s from three independent experiments. * and NS denote *P*<0.05 and *P*>0.05, respectively. Numbers above bars present the fold changes *versus* the indicated control values. (**f**) Proteins from transfected A549 or H1299 cells were electrophoresed and subjected to OxyBlot analysis for detecting carbonylated proteins. (**g**) *γ*-H2AX levels in A549 or H1299 cells were immunoblotted to evaluate the extent of DNA breakage. ARD1 levels were also checked in the same samples to verify overexpression and knockdown of ARD1

To obtain further insight into the role of ARD1 in oxidative stress, we measured basal levels of intracellular ROS. As expected, the number of A549 cells emitting DCF fluorescence was decreased by MSRA overexpression and increased by MSRA knockdown (Supplementary Figure S3a). When DCF fluorescence was analyzed by flow cytometry, the population of DCF fluorescence-positive A549 cells noticeably increased under hydrogen peroxide (H_2_O_2_) stimulation; it was suppressed by MSRA overexpression but augmented by MSRA knockdown (Supplementary Figures S3b and S3c). These results confirm the antioxidant action of MSRA. Having shown that ARD1 inhibits MSRA function, we expected ARD1 to have a pro-oxidant role during the oxidative stress. The pro-oxidant role of ARD1 was clearly shown in three cell lines (Figure 4a) and was confirmed by flow cytometry (Figure 4b). Total fluorescence intensities were calculated and presented as relative levels to the vector control value. ROS levels inversely correlated with ARD1 expression in both unstimulated and H_2_O_2_-stimulated A549 cells and were not affected by ARD1 in MSRA-deficient cells (Figures 4c and d). As was expected, ROS levels were substantially reduced by either wild-type or K49R-mutated MSRA overexpression, and were attenuated by ARD1 coexpression (Figure 4e). However, the antioxidant effect of K49R-mutated MSRA, *versus* wild-type MSRA (4.1-fold increase of ROS), was inhibited to a lesser extent (1.9-fold) by ARD1 coexpression. These results suggest that ARD1 increases intracellular ROS levels by acetylating and inactivating MSRA. We next examined whether ARD1 determines the cellular response to oxidative stress. Protein carbonylation, which results from protein oxidation,^21,22^ occurred basally in unstimulated A549 and H1299 cells, and was profoundly increased by H_2_O_2_. The carbonylation was found to be associated with ARD1 expression (Figure 4f). Moreover, the expression of *γ*-H2AX, which is known to be induced by ROS-mediated DNA breakage,^23,24^ was positively regulated by ARD1 (Figure 4g). This suggests that ARD1 has a pro-oxidant role under both resting and oxidative conditions.

### ARD1 promotes necrotic cell death under oxidative stress

**Figure 5:**
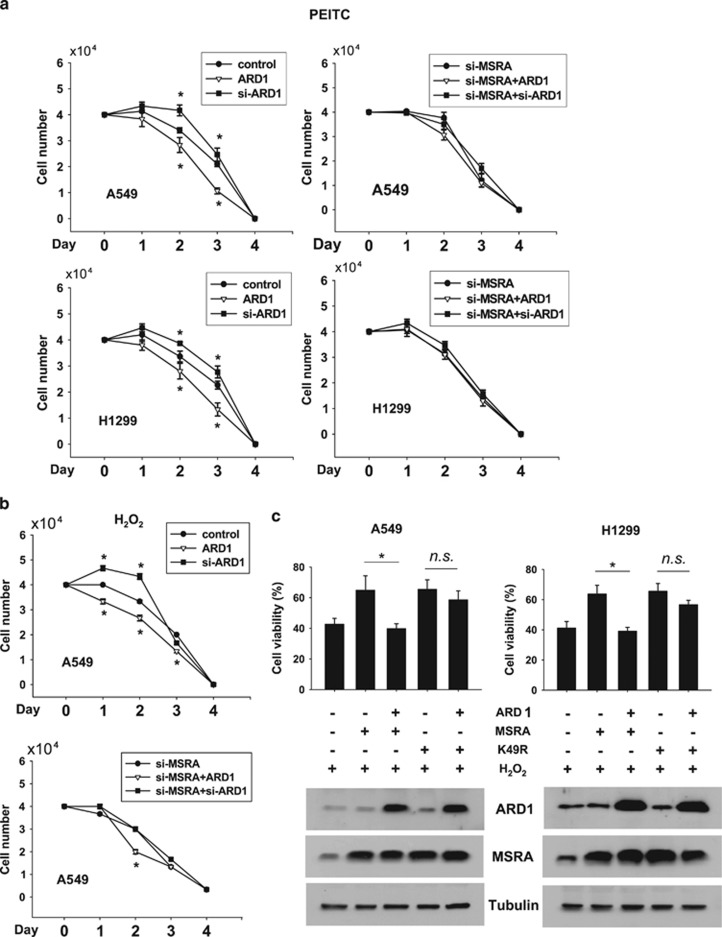
ARD1 facilitates cell death under oxidative stress. (**a**) A549 or H1299 cells, which had been transfected with ARD1 plasmid or siRNA, were plated in 24-well cell culture dishes (4 × 10^4^ per well) and treated with 10 *μ*M PEITC the next day. After being incubated for the indicated time, cells unstained with trypan blue were counted as viable cells. (**b**) Transfected A549 cells (4 × 10^4^ per well) were treated with 40 *μ*M H_2_O_2_ and incubated for the indicated time. Viable cells were counted as described above. Results are shown as means±S.D.s from three independent experiments and *denotes *P*<0.05 *versus* the control value in the same incubation time. (**c**) A549 or H1299 cells, which had been transfected as indicated, were treated with 40 *μ*M H_2_O_2_ for 48 h, and cell viability was measured using MTT. Results are shown as means+S.D.s from three independent experiments. * and NS denote *P*<0.05 and *P*>0.05, respectively

**Figure 6:**
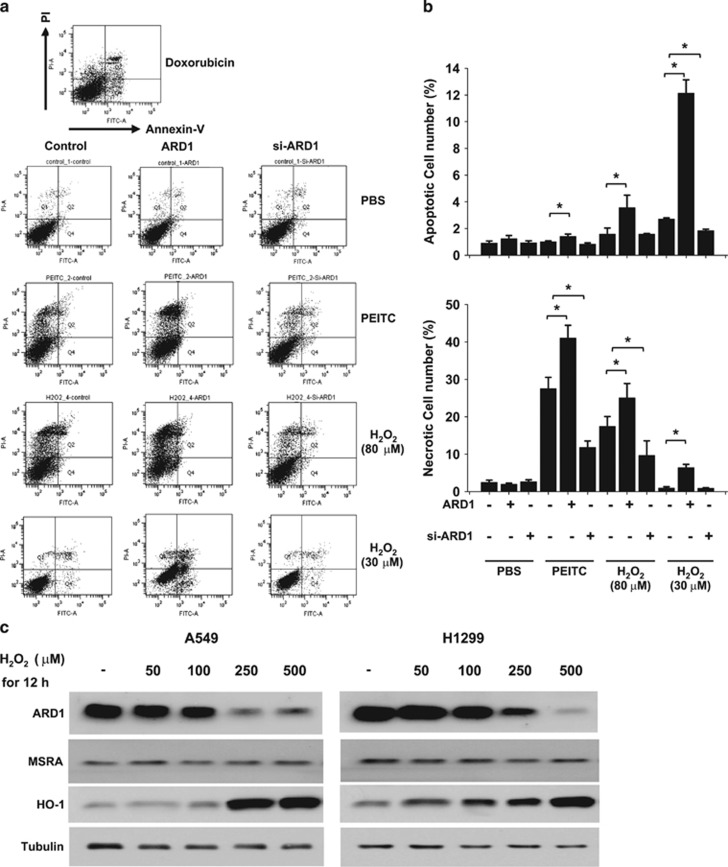
ARD1 facilitates either necrotic or apoptotic cell death under oxidative stress. (**a**) Transfected A549 cells were treated with PBS, 20 *μ*M PEITC, 80 *μ*M H_2_O_2_, 30 *μ*M H_2_O_2_ or 0.5 *μ*M doxorubicin for 24 h. Cells were stained with annexin-V and PI and subjected to flow cytometric analyses. Cells in the upper left quadrant (annexin-V negative and PI positive) are considered to be necrotic and cells in the lower right quadrant (annexin-V positive and PI negative) to be apoptotic. (**b**) The number of cells in the above histograms were counted and plotted as bar graphs. Each bar represents the mean+S.D. from three independent experiments and *denotes *P*<0.05 between the two groups. (**c**) A549 or H1299 cells were incubated with the indicated concentrations of H_2_O_2_ for 12 h, and subjected to western blotting. Heme oxygenase 1 was measured as a positive control reflecting the cellular response to oxidative stress

We next assessed the possibility that ARD1 is involved in cell death under oxidative stress. We treated cells with the oxidative stressors H_2_O_2_ and phenethyl isothiocyanate (PEITC)^25^ and analyzed cell viability by counting cells not stained with trypan blue or by MTT staining. Both oxidative stressors induced death of A549 or H1299 cells in a time-dependent manner. Cell death was augmented by ARD1 overexpression and attenuated by ARD1 knockdown. In MSRA-deficient cells, however, oxidative stress-induced cell death was not or was less affected by ARD1 (Figures 5a and b). To examine whether the ARD1's effect is specific to oxidative cell death, we induced cell death using a DNA damaging agent cisplatin.^26^ Cisplatin induced cell death constantly regardless of ARD1 and MSRA expression (Supplementary Figure S4). We performed MTT analysis as another index for cell death, and also found that ARD1 augments oxidative cells and the effect of ARD1 is less significant in MSRA-deficient cells (Supplementary Figure S5). When MSRA was overexpressed in A549 and H1299, both cell lines became resistant to H_2_O_2_, which was attenuated by ARD1 coexpression. In contrast, both cell lines expressing K49R-mutated MSRA kept the resistance even with ARD1 coexpression (Figure 5c). To further understand how ARD1 promotes oxidative cell death, we performed annexin-V/propidium iodide (PI) double staining followed by flow cytometry, and verified this method using doxorubicin as a representative cytotoxic agent (Figure 6a, the first row). As shown in Figure 6a, the population of necrotic cells (upper left quadrant) was profoundly increased by 20 *μ*M PEITC or 80 *μ*M H_2_O_2_ treatment compared with that of apoptotic cells (lower right quadrant). The number of cells undergoing necrosis appeared to be dependent on the ARD1 level (Figure 6a, the third and fourth rows). When A549 cells were treated with 30 *μ*M H_2_O_2_, the proportion of apoptosis was relatively increased by ARD1 overexpression (Figure 6a, the fifth row). We counted cells in four windows and confirmed that the majority of cells underwent necrosis under severe oxidative stress or underwent apoptosis under mild stress and that both types of cell death were induced depending on ARD1 (Figure 6b). Therefore, ARD1 is likely to promote cell death under oxidative stress by inhibiting the antioxidant function of MSRA. Then, what is the physiological meaning of the ARD1-dependent regulation of MSRA? To answer this question, we first tested the possibility that ARD1 expression is regulated to keep the redox homeostasis. Interestingly, ARD1 expression in A549 and H1299 cells was repressed under H_2_O_2_ stress in a concentration-dependent manner, whereas MSRA was consistently expressed irrespective of oxidative stress (Figure 6c). Reversely, heme oxygenase 1, which is an antioxidant enzyme, was induced by H_2_O_2_, supporting that the cells normally respond to oxidative stress. To adapt to oxidative stress, cells sorely need the antioxidant action of MSRA and the ARD1 suppression may meet this requirement.

### ARD1 transgenic mice are more susceptible to oxidative stress

**Figure 7:**
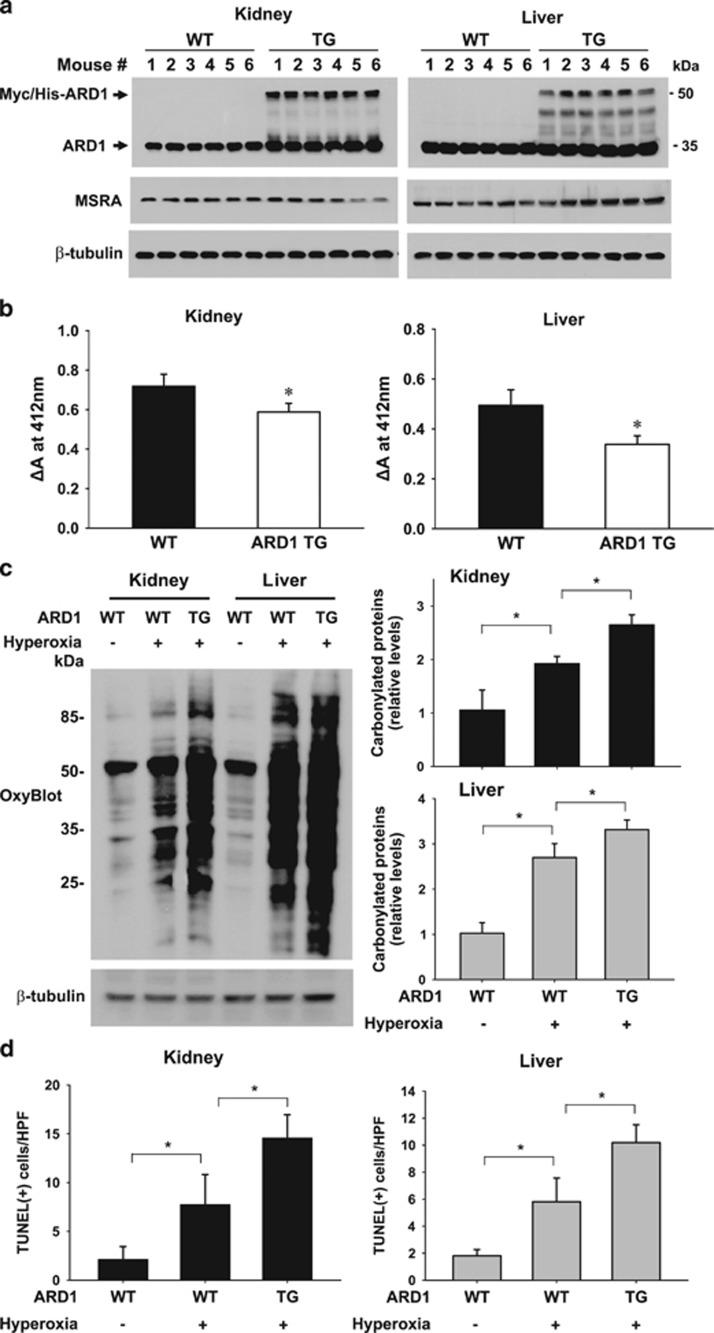
ARD1 transgenic mice are susceptible to oxidative stress. (**a**) Transgenic Myc/His-ARD1, endogenous ARD1 and MSRA proteins were immunoblotted in kidney and liver homogenates from transgenic (TG) mice and their wild-type (WT) littermates. (**b**) MSRA activities were analyzed in kidney and liver homogenates from TG mice and WT littermates. Polytronized homogenates were centrifuged at 10 000 g at 4 °C for 30 min and the supernatants (finally 500 *μ*g of proteins) were applied to the MSRA assay system at 37 °C for 30 min. MSRA activity was accessed by monitoring the absorbance change (ΔA) at 412 nm. Each bar represents the mean+S.D. (*n*=6 in each group) and *denotes *P*<0.05 between the two groups. (**c**) Representative OxyBlot images of kidney and liver homogenates from wild-type mice and ARD1 transgenic mice. Carbonylated proteins were derivatized with 2,4-DNP and were immunoblotted, which was quantified using the ImageJ program (The National Institutes of Health, Bethesda, MD, USA). Results are shown as the mean+S.D.s from six samples per each group. *P<0.05. (**d**) The number of TUNEL-positive cells (per high power field) in kidney and liver tissues were counted in four consecutive fields. Results are shown as the mean+S.D.s from six samples. *P<0.05

To induce oxidative stress *in vivo*, mice were incubated in a normobaric, hyperoxic (100% oxygen) chamber. Also, to gain further insight into the role of ARD1 in oxidative injury, we carried out the experiment using ARD1 transgenic mice. Transgenic Myc/His-ARD1 was expressed in the kidneys and livers of the transgenic mice (Figure 7a). Despite the absence of changes in MSRA levels between the two groups (Figure 7a), MSRA activities in kidney and liver tissues decreased significantly in ARD1 transgenic mice (Figure 7b), which further supports the inhibitory role of ARD1 in MSRA function. ARD1 transgenic mice and their wild-type littermates were exposed to normoxia or hyperoxia for 2 days, and then carbonylated proteins were analyzed in kidney and liver homogenates. Many proteins from the kidney and liver tissues were shown to be more highly carbonylated by hyperoxic stress, and the extent of carbonylation was augmented in ARD transgenic mice compared with wild-type mice (Figure 7c, left panel). Significant differences in protein carbonylation were shown in densitometric analyses (Figure 7c, right panel). To evaluate tissue injury induced by hyperoxygenation, a TUNEL assay was performed. More TUNEL-positive cells were detected in kidneys and livers from ARD1 transgenic mice than in those from wild-type mice, although no differences were found among the groups in terms of H&E staining (Supplementary Figure S6a). Histological examination showed significant differences in the numbers of TUNEL-positive cells (Figure 7d and Supplementary Figure S6b). These results further support our notion that ARD1 promotes oxidative injury.

## Discussion

Given that MSRA was hunted with the bait ARD1 in the yeast two-hybrid screen, we began to explore a new role of ARD1 as an upstream regulator of MSRA. Cell-based and *in vitro* data strongly indicate that ARD1 directly binds to MSRA, acetylates it at K49 and inhibits its enzymatic function. As a consequence of MSRA inhibition, ARD1 increases the levels of ROS, carbonylated proteins and DNA breaks under oxidative stress, thereby promoting cell death. In addition, the livers and kidneys of ARD1 transgenic mice were more vulnerable to oxygen toxicity. ARD1 may be a bona fide regulator of MSRA that favors the oxidative state, and is a potential target for modulating oxidative injury.

The MSRA protein consists of three domains: an N-terminal mitochondrial targeting sequence domain, a catalytic cysteine domain and a C-terminal thioredoxin-binding domain. There are two MSRA transcript variants: a long form containing 235 amino acids and a short form containing 192 amino acids in which the N-terminus is deleted. The long form of MSRA localizes to mitochondria, the nucleus and the cytoplasm, whereas the short form is present in the nucleus and cytoplasm.^27^ We here found that the Lys49 residue of MSRA is acetylated by ARD1. Given that Lys49 is conserved in the catalytic domain of both variants, it is expected that the enzymatic activities of both MSRA variants are modulated by ARD1-mediated Lys49 acetylation. Indeed, our *in vitro* and *in vivo* results strongly suggest that ARD1 negatively regulates the total activity of MSRA.

The enzymatic activity of MSRA can be divided into three steps: formation of a sulfenic acid intermediate from MetO, formation of an intramolecular disulfide bond, and reduction of the disulfide bond by thioredoxin.^28^ Because the last step can be catalyzed by DTT instead of thioredoxin, we can measure MSRA activity using DTT and the chromogenic substrate dithio-bis-nitrobenzoic acid (DTNB, alternatively named Ellman's reagent).^29^ Compared with the conventional HPLC method of direct detection of methionine derivatives, this enzymatic method is easier and more rapid. When we traced the absorbance at 412 nm using this method, we found that MSRA activity was reduced by ARD1-mediated Lys49 acetylation. To our knowledge, such regulation of MSRA is suggested for the first time in this study.

MSRA functions to recover sulfoxidized methionine.^30^ Methionine is not only an amino acid essential for peptide synthesis but also delivers the methyl moiety as a form of *S*-adenosylmethionine.^31^ Oxidative stress oxidizes methionine to MetO, which decreases the intracellular level of free methionine and subsequently impairs peptide synthesis and many methylation processes.^14^ Also, the sulfoxidation of methionine residues alters the conformation and function of proteins.^32^ Therefore, MSRA has a key role in protecting cells from oxidative injury.^33, 34, 35^ MSRA is also known to regulate redox-dependent signaling pathways. For example, calcium-calmodulin-dependent protein kinase II is activated ROS-dependently via the sulfoxidation of Met281 and Met282, which is reversed by MSRA.^36^ Besides these functions, MSRA decreases production of ROS by repairing antioxidant enzymes whose methionine residues are sulfoxidized.^37^ Considering these diverse actions of MSRA against oxidative stress, it is not surprising that the MSRA inhibitor ARD1 increases ROS levels and augments oxidative cell death.

MSRA knockout mice have a shorter lifespan in a hyperoxic chamber^38^ and are more susceptible to ischemia–reperfusion injury in the kidney.^39^ Given that hyperoxia and ischemia–reperfusion are representative modalities for inducing oxidative stress, the *in vivo* evidence strongly supports the protective role of MSRA against oxidative stress. These reports encouraged us to examine *in vivo* the role of ARD1 in MSRA's action against oxidative stress. We found that protein carbonylation under hyperoxic stress was more severe in ARD1 transgenic mice than in their wild-type littermates. Next, we performed a TUNEL assay to evaluate the role of ARD1 in hyperoxygenation-induced tissue injury and found that dying cells in the kidney and liver are more abundant in ARD1 transgenic mice than in their wild-type littermates. Although TUNEL detects DNA fragmentation, which is a hallmark of apoptosis, recent reports have demonstrated that *in situ* TUNEL assays cannot distinguish among the features of cell death by apoptosis, necrosis and autophagy.^40^ To further evaluate the features of cell death induced by oxidative stress, we costained cells with annexin-V and PI as apoptosis and necrosis markers, respectively. We found that necrotic and apoptotic cells coexist under oxidative stress induced by either PEITC or H_2_O_2_. Quantitative analyses of FACS data revealed that necrotic cells were more abundant than apoptotic cells and that necrotic and apoptotic cell numbers were increased by ARD1 overexpression. ARD1 is likely to promote oxidative injury, irrespective of cell death features.

We here found that ARD1 is downregulated during oxidative stress. To date, the regulation of ARD1 expression has not been intensively explored. Only a few reports suggested that a HIF-1 inhibitor ARD1 is downregulated during hypoxia and this ensures the HIF-1-mediated adaptation to hypoxia.^41^ Although such a role of ARD1 has been seriously questioned by other investigators,^42^ it is noted that the hypoxic suppression of ARD1 should not be ignored. Indeed, we previously observed the ARD1 suppression by hypoxia and empirically knew this regulation of ARD1 was variable depending on cell contexts (data not shown). In our opinion, the ARD1 suppression under hypoxia may be related with the ARD1 suppression under oxidative stress because, ironically, intracellular ROS levels increase during hypoxia.^43^ The precise mechanism underlying ARD1 regulation is an open question.

This study shows that ARD1 negatively controls the enzymatic activity of MSRA by acetylating it at K49 and by doing so shifts the intracellular redox balance toward a more oxidative state. To understand the role of ARD1 in the cellular response to oxidative stress, we carried out most experiments under harsh conditions and found that ARD1 promotes oxidative injury. Considering the Janus-faced role of ROS, however, it is unclear whether or not ARD1 has distinct roles in ROS-mediated cell signaling and proliferation, and this point is an open question. In conclusion, ARD1 was newly shown in this study to have a pro-oxidant role as a negative regulator of MSRA.

## Materials and Methods

### Reagents and antibodies

Anti-ARD1 antibody was generated in rats against full-length human ARD1 peptide. Antibodies against MSRA, acetylated lysine, *β*-tubulin and FLAG tag were purchased from Novus Biologicals (Littleton, CO, USA), Cell Signaling (Danvers, MA, USA), Santa Cruz Biotechnology (Santa Cruz, CA, USA) and Sigma–Aldrich (St. Louis, MO, USA), respectively. Fetal bovine serum (FBS), dithiothreitol (DTT), 5,5′-DTNB, L-MetO, dimethyl sulfoxide, doxorubicin, PEITC, 2′,7′-dichlorofluorescin diacetate (DCF-DA) and others were obtained from Sigma–Aldrich.

### siRNAs and plasmids

The nucleotide sequences (5′ to 3′) of ARD1 (NM_003491), MSRA (NM_012331) and non-targeting siRNA were 5′-CGAGCUUUCACAAUAAAUUUGCUCC-3′, 5′-GGGACAGACUUUCUACUAUGCGGAA-3′ and 5′-AUGAACGUGAAUUGCUCAA-3′, respectively. The cDNAs of ARD1 and MSRA were cloned by reverse transcription and PCR using Pfu DNA polymerase, and the cDNAs were inserted into pcDNA or Flag-tagged pcDNA vector by blunt-end ligation. MSRA-K49R was made by substituting Arg for Lys49 in MSRA, using a Quick- Change site-directed mutagenesis kit (Stratagene, Cedar Creek, TX, USA).

### Cell lines and cell culture

HEK293T (human embryonic kidney), H1299 (human lung carcinoma) and A549 (human lung carcinoma) cell lines were obtained from the American Type Culture Collection (Manassas, VA, USA), and cultured in RPMI1640 or DMEM supplemented with 10% heat-inactivated FBS in a 5% CO_2_ humidified atmosphere at 37 °C.

### Animals

Myc/His-tagged human ARD1 transgenic mouse was generated using the CAGGS expression vector containing cytomegalovirus enhancer fused to the chicken *β*-actin promoter. The transgenic DNA construct was microinjected into the fertilized eggs collected from C57BL/6 mice, as previously described.^44^ Genomic DNAs were obtained from the tails of founder mice at 2 weeks of age, and genotyping was performed by PCR and Southern blotting. Mice were bred in the hemizygous state, and transgenic mice and non-transgenic littermates were assigned to pair-matched groups for all experiments. Mice were bled and maintained in Ewha Laboratory Animal Genomics Center under specific pathogen-free conditions. To induce oxidative stress, mice were put into an air-tight chamber where humidified air (normoxia) or 100% O_2_ (hyperoxia) gas was continuously supplied for 2 days. As soon as the mice were taken out, they were decapitated for tissue preparation. After briefly rinsing with cold PBS, tissues were quickly frozen in liquid nitrogen for protein carbonylation assay or fixed in 10% formalin for TUNEL and histological examinations. All experiments were approved by the Institutional Animal Care and Use Committees of Ewha Women's University and adhered to the National Research Council Guidelines (approve # 2010-24-2).

### Immunoblotting and immunoprecipitation

The cell lysates were separated on SDS-polyacrylamide gels and transferred to Immobilon-P membranes (Millipore; Bedford, MA, USA). Membranes were blocked with a Tris/saline solution containing 5% skim milk and 0.1% Tween-20 for 1 h and incubated with a primary antibody overnight at 4 °C. Membranes were incubated with a horseradish peroxidase-conjugated secondary antibody for 1 h and visualized using the ECL kit (Thermo, Rockford, IL, USA). To analyze protein interactions, the cell lysates were incubated with anti-ARD1, anti-Flag or anti-acetylated lysine (Ac-K) antibody for 4 h at 4 °C, and the immune complexes were precipitated with protein A/G beads (Santa Cruz, CA, USA). Precipitated proteins were eluted in a denaturing SDS sample buffer, loaded on SDS-PAGE and immunoblotted.

### *In vitro* binding and acetylation assays

His-tagged ARD1 (His-ARD1) or glutathione S-transferase-tagged MSRA (GST-MSRA) peptide was expressed with IPTG in BL21 *Escherichia coli* and purified using nickel- or glutathione-affinity beads. The purities (>95%) of recombinant peptides were verified by SDS-PAGE and Coomassie Blue R-250 staining. For protein binding assays, 1 mg of GST-MSRA and 0.5 mg of His-ARD1 were incubated in a binding buffer (25 mM HEPES, pH 7.5, 150 mM KCl, 12.5 mM MgCl_2_, 20 *μ*M ZnCl_2_, 5 mM DTT, 0.1% NP40 and 10% glycerol) at 4 °C for 4 h. Proteins were pulled down with glutathione-affinity beads and identified by immunoblotting and Coomassie Blue staining. For *in vitro* acetylation, 0.5 mg of His-ARD1 and 1 mg of GST-MSRA were incubated in a reaction mixture containing 50 mM Tris/HCl (pH 8.0), 0.1 mM EDTA, 1 mM DTT, 10 mM sodium butyrate, 2 mM Acetyl-CoA and 10% glycerol for 3 h. Acetylated GST-MSRA peptides were identified by immunoblotting with anti-Ac-K antibody.

### In-gel digestion and mass spectrometric analysis

GST-MSRA peptides subjected to the *in vitro* acetylation assay were electrophoresed on SDS-PAGE, and the gel pieces including GST-MSRA were cut out and digested with trypsin. Peptide fragments were subjected to liquid chromatography/MASS analysis (Q-tof Ultra global), which was equipped with a three-pumping Waters nano-LC system, a stream selection module and MassLynx 4.0 controller (Waters, UK). Briefly, 5 *μ*l of samples was dissolved in buffer C (95 : 5 : 0.2, water/CAN/formic acid, v/v/v), injected on a column and eluted by a linear gradient of 5–80% buffer B (95 : 5 : 0.2, ACN/water/formic acid, v/v/v) over 120 min. MS/MS spectra were processed and subjected to database searches in SWISS-PROT or Mascot database using ProteinLynx Global Server (PLGS) 2.1 software (Micromass, UK). Acetylation was identified by the additional mass of 42 Da on lysine residue.

### MSRA activity assay using the colorimetric method

The enzymatic activity of MSRA was measured in a reaction buffer (25 mM Tris/HCl, pH 8.0, 10 mM MgCl_2_, 30 mM KCl, 0.1 mM DTT and 0.25 mM MetO). Recombinant MSRA peptides, cell lysates or tissue homogenates were added to the reaction solution. The reaction was carried out at 37 °C for 30 min in the dark. To detect the remaining MetO, an equal volume of 4 mM DTNB was added to the reaction solution, and further incubated at 37 °C for 10 min. The enzymatic reaction was checked by recording the absorbance change at 412 nm in a UV/VIS-spectrophotometer, as described previously.^29^

### Cell viability assay

Cells were grown in 98-well culture plates. After being treated with doxorubicin, H_2_O_2_ and PEITC for 24 h, the cells were incubated with 100 *μ*l of MTT labeling reagent (Sigma–Aldrich) for 3 h. Blue formazan crystals were solubilized with acidified isopropanol, and formazan levels were determined at 570 nm.

### ROS measurement

As soon as culture plates were displaced from an incubator, DCF-DA (20 *μ*M) was loaded onto cells. After being incubated in the dark for 20 min, cells were detached in a trysin-EDTA solution, spun down and washed twice in PBS. Cell pellets were resuspended in PBS and applied to a BD FACS Canto Flow Cytometer (Becton Dickinson and Company, BD Biosciences, CA, USA). DCF fluorescence was exited at 488 nm and detected at 524 nm.

### Protein carbonylation analysis

Protein carbonylation was evaluated using the OxyBlot Protein Oxidation Detection kit (EMD Millipore Corporation, Billerica, MA, USA). Briefly, 2,4-dinitrophenylhydrazine was added to whole-cell extracts to derivatize the carbonyl groups in the protein side chains to 2,4-dinitrophenylhydrazone (DNP), which was then revealed by western blotting with anti-DNP antibodies.

### Analysis of cell death

Cell death was analyzed using the Annexin V FITC apoptosis detection kit (BD Biosciences) and flow cytometry. Briefly, cells were resuspended in a binding buffer and incubated with annexin-V FITC for 15 min in the dark at room temperature and then treated with PI. Flow cytometric analysis was immediately performed in a BD FACS Canto Flow Cytometer.

### TUNEL assay

The Apoptag *In Situ* Apoptosis Detection Kit (Oncor, Gaithersburg, MD, USA) was used to identify dead cells in mouse tissues. Fixed tissue sections were deparaffinized, rehydrated, treated with proteinase K and incubated with a TdT enzyme solution at 37 °C for 2 h. The reaction was carried out at 37 °C for 30 min and terminated in a stop and wash buffer provided by the supplier. The sections were incubated with anti-digoxigenin peroxidase (130 *μ*l per 10 cm^2^) and then with diaminobenzidine (150 *μ*l per 10 cm^2^) containing 0.01% H_2_O_2_ for 5 min. Finally, the sections were lightly counterstained with H&E and examined under a microscope.

### Statistics

All data were analyzed using Microsoft Excel 2013 software (Microsoft Korea Co., Seoul, Korea), and results are expressed as means and S.D. We used the unpaired, two-sided Student *t*-test to compare DCF fluorescence, light absorption and cell numbers. Statistical significances were considered when *P*-values were <0.05.

## Abbreviations

Ac-K: acetylated lysine
ARD1: arrest defective 1
MetO: methionine sulfoxide
MSRA: methionine sulfoxide reductase A
PEITC: phenethyl isothiocyanate
ROS: reactive oxygen species
