# Supplementary figures and images for: Arrest defective 1 regulates the oxidative stress response in human cells and mice by acetylating methionine sulfoxide reductase A
Source: Cell Death Dis. 2014 Oct 23;5(10):e1490–. doi: 10.1038/cddis.2014.456 (PMC4649535; doi:10.1038/cddis.2014.456)

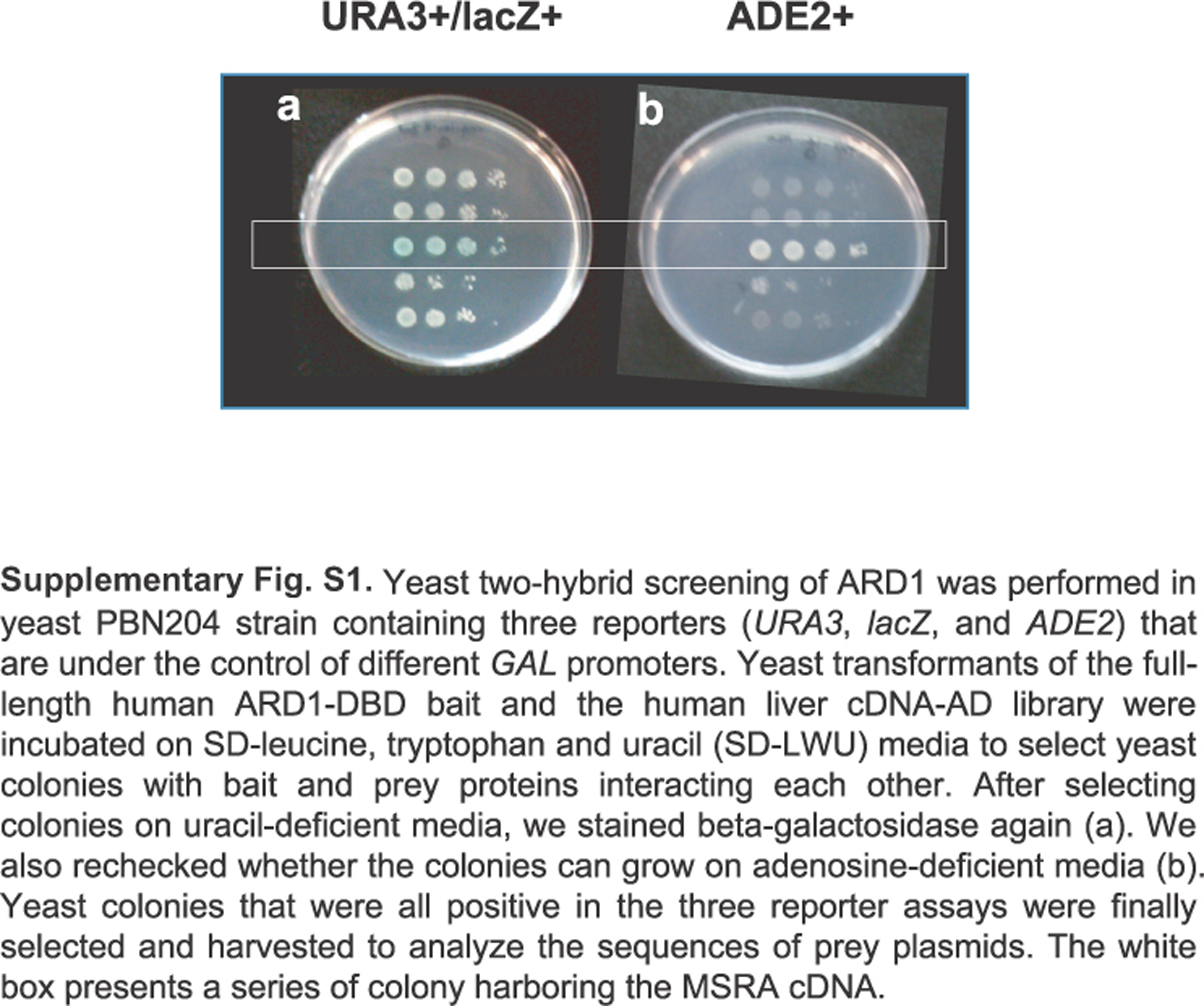

Supplement: Supplementary Figure 1 [file cddis2014456x1.tif]

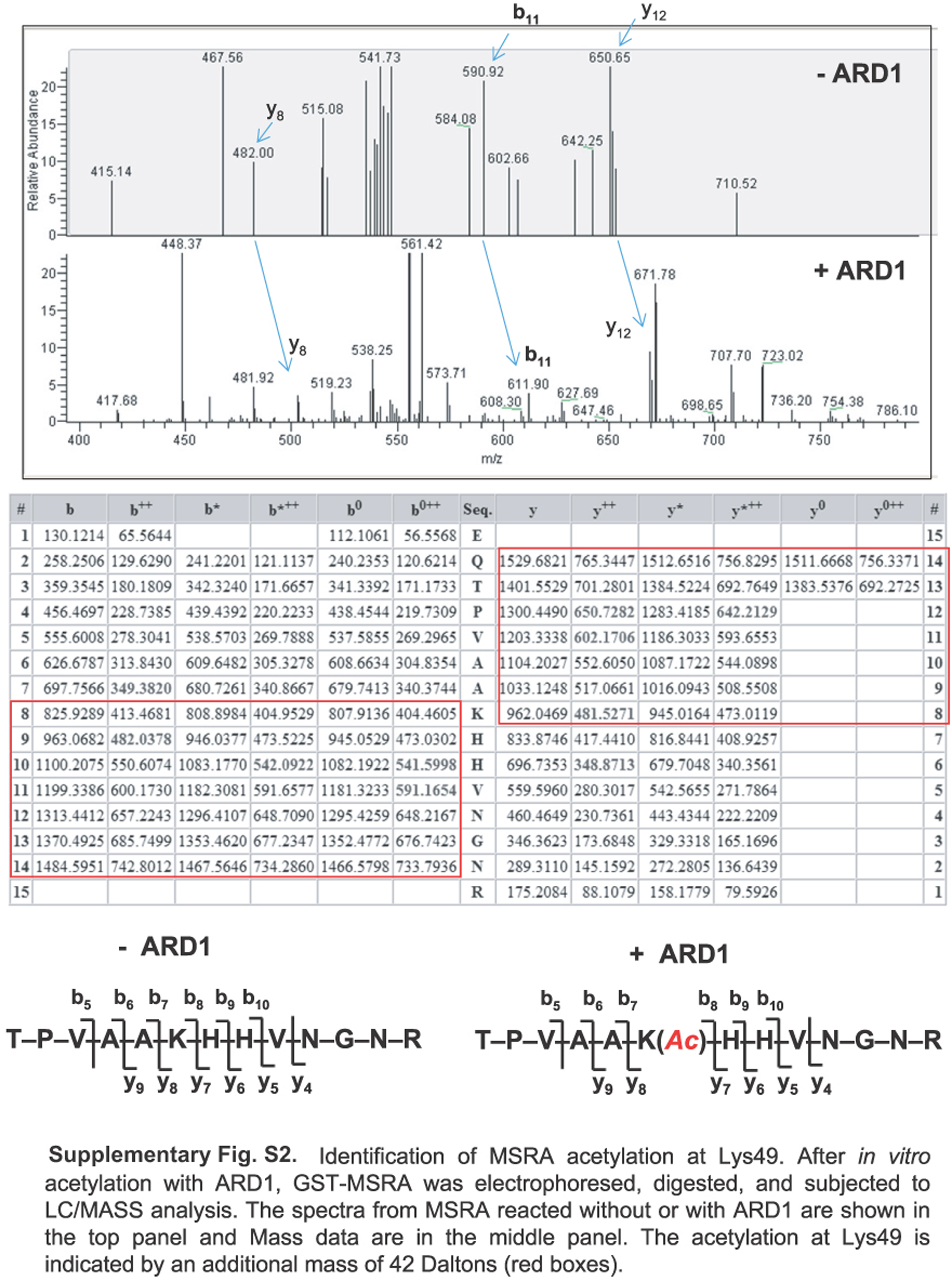

Supplement: Supplementary Figure 2 [file cddis2014456x2.tif]

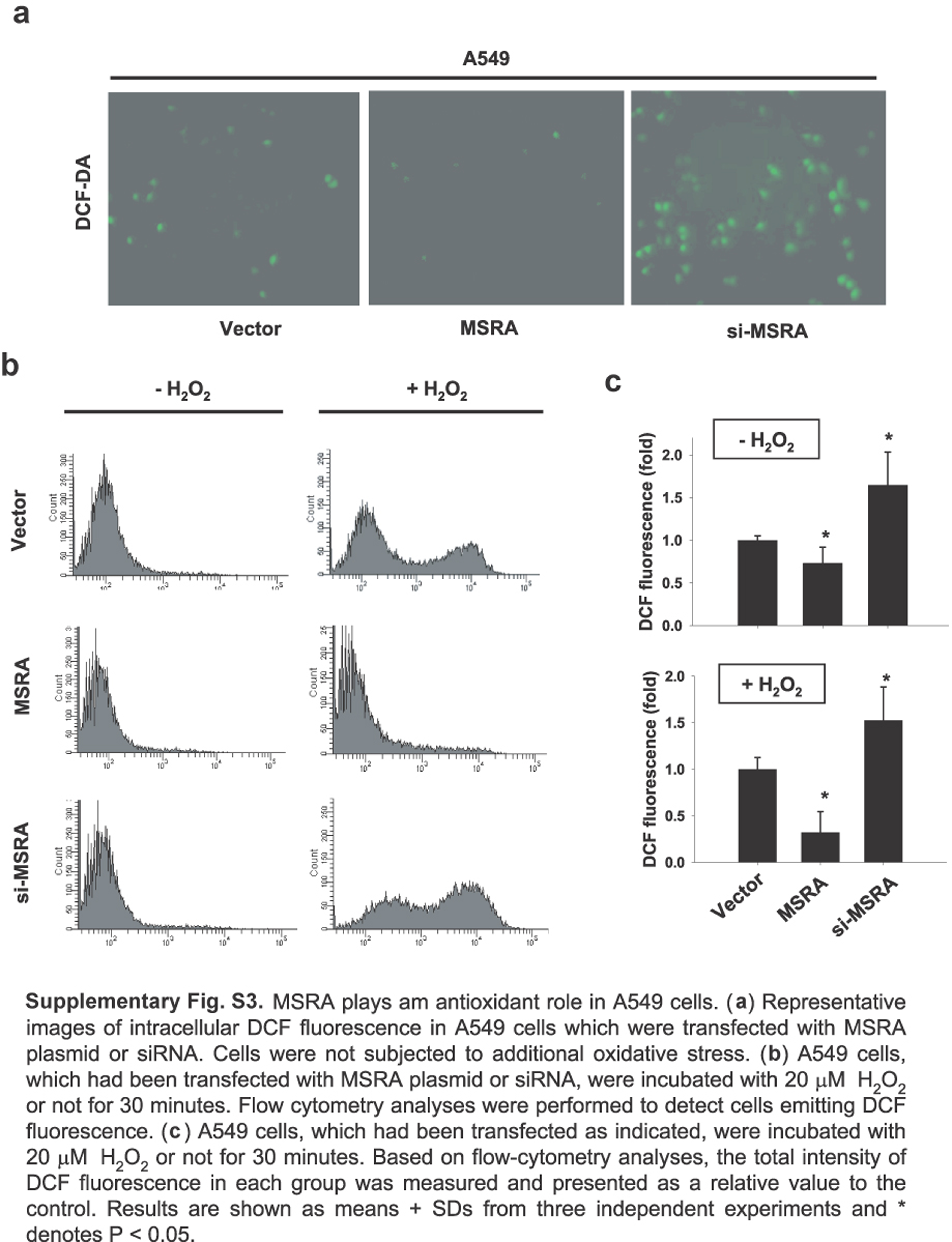

Supplement: Supplementary Figure 3 [file cddis2014456x3.tif]

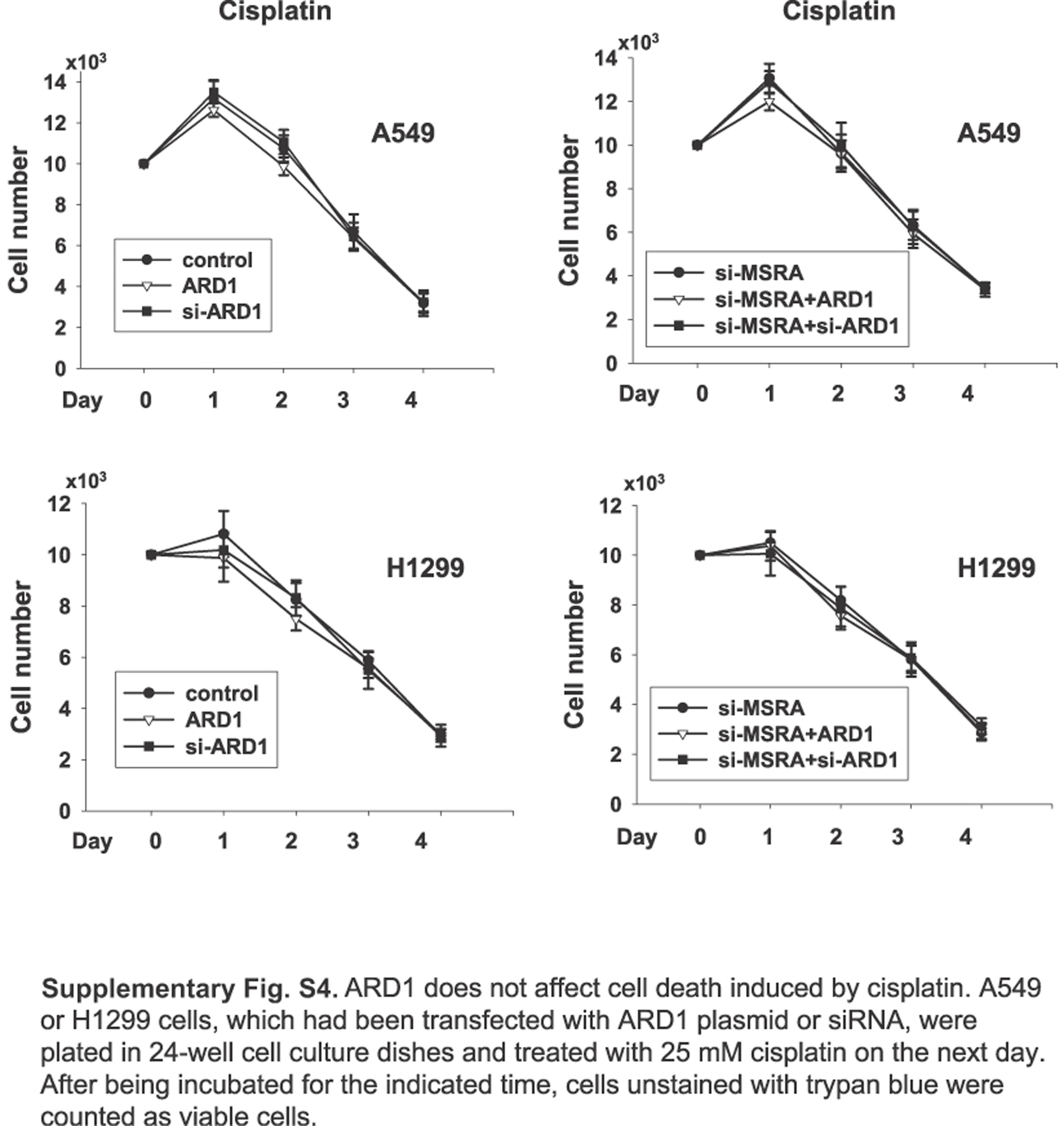

Supplement: Supplementary Figure 4 [file cddis2014456x4.tif]

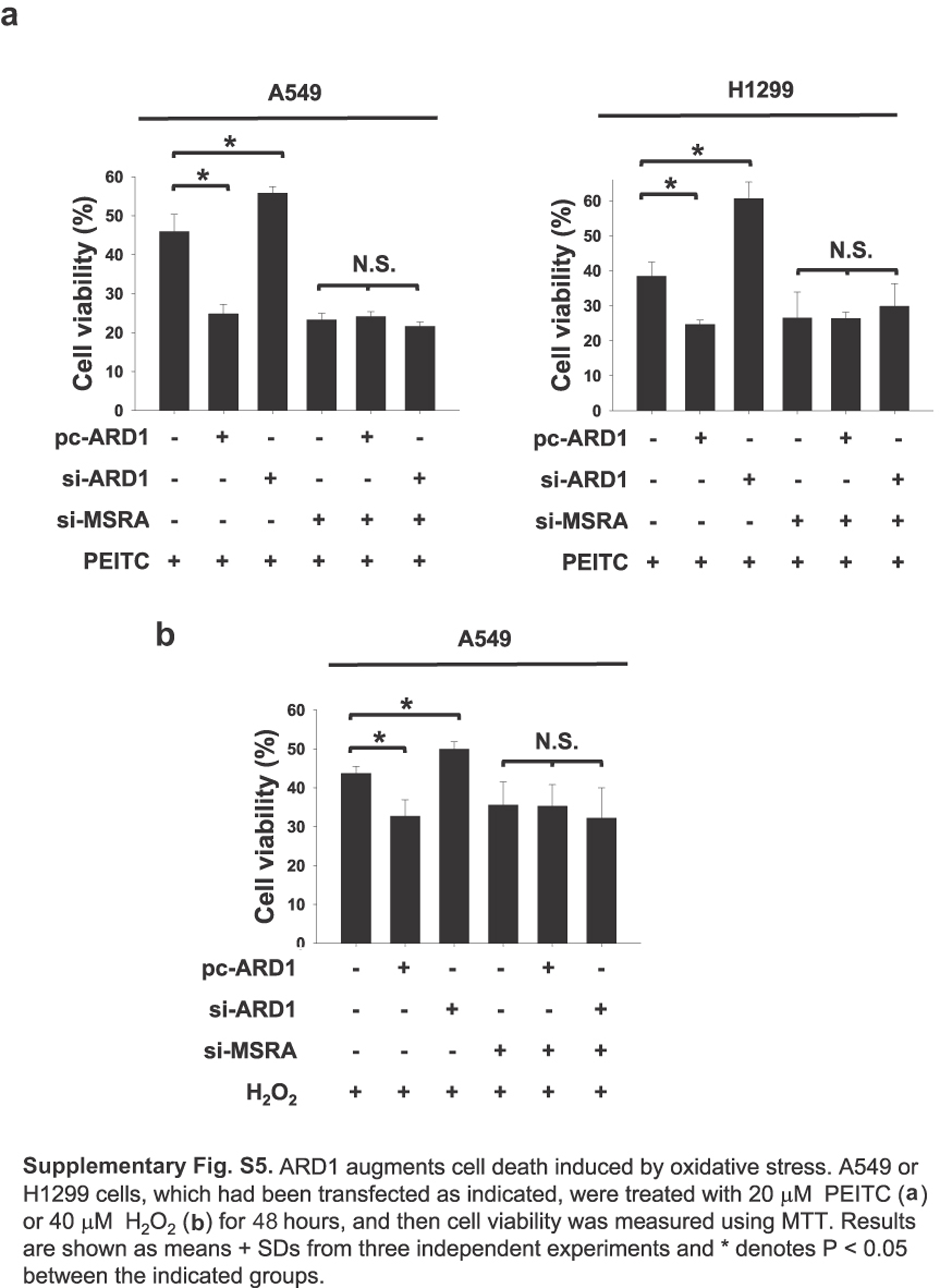

Supplement: Supplementary Figure 5 [file cddis2014456x5.tif]

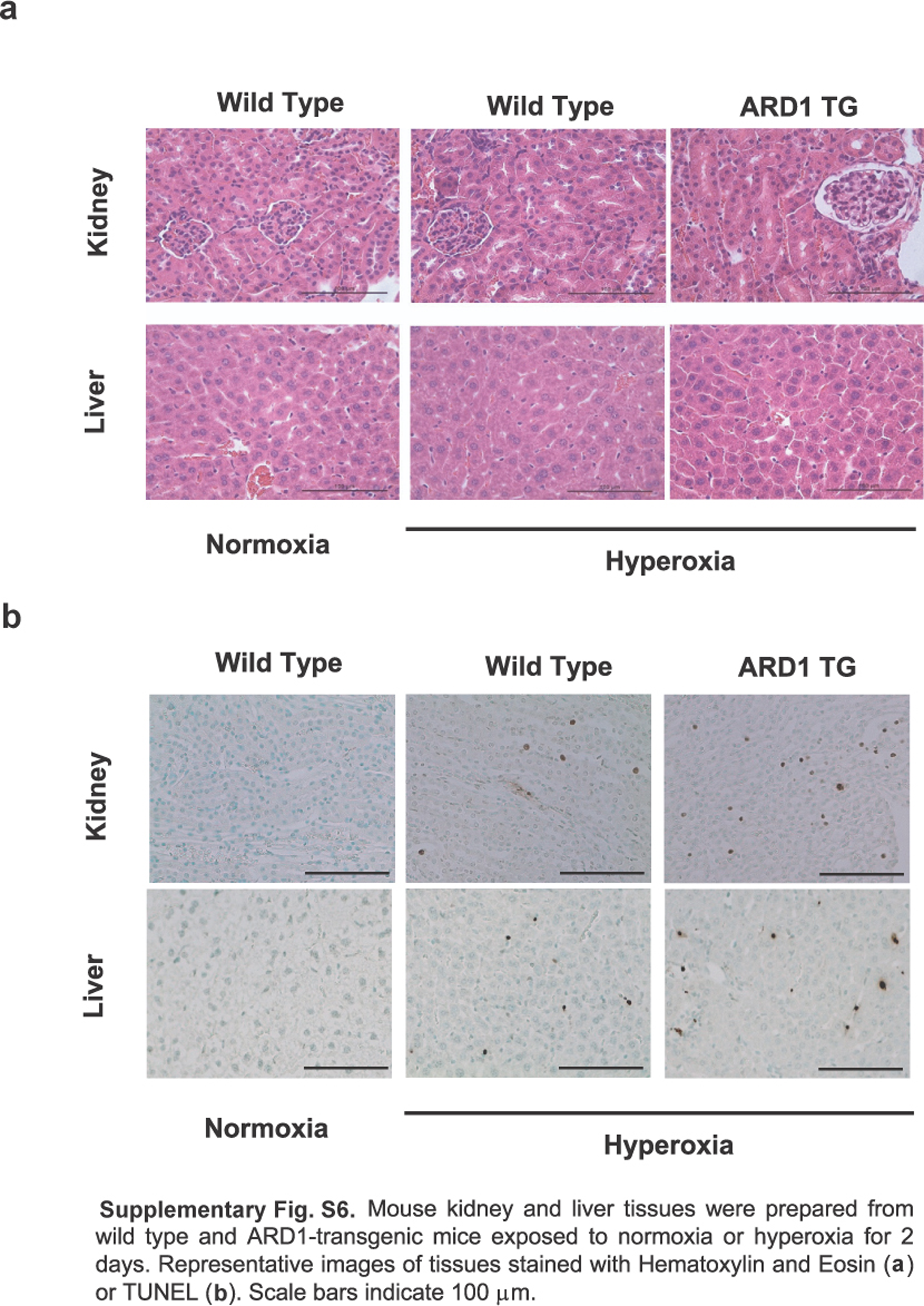

Supplement: Supplementary Figure 6 [file cddis2014456x6.tif]
